# Efficient Communication in Distributed Simulations of Spiking Neuronal Networks With Gap Junctions

**DOI:** 10.3389/fninf.2020.00012

**Published:** 2020-05-05

**Authors:** Jakob Jordan, Moritz Helias, Markus Diesmann, Susanne Kunkel

**Affiliations:** ^1^Department of Physiology, University of Bern, Bern, Switzerland; ^2^Institute of Neuroscience and Medicine (INM-6), Jülich Research Centre, Jülich, Germany; ^3^Institute for Advanced Simulation (IAS-6), Jülich Research Centre, Jülich, Germany; ^4^JARA Institute Brain Structure Function Relationship (INM-10), Jülich Research Centre, Jülich, Germany; ^5^Department of Physics, Faculty 1, RWTH Aachen University, Aachen, Germany; ^6^Department of Psychiatry, Psychotherapy and Psychosomatics, Medical Faculty, RWTH Aachen University, Aachen, Germany; ^7^Faculty of Science and Technology, Norwegian University of Life Sciences, Ås, Norway

**Keywords:** gap junctions, electrical synapses, spiking neuronal network, large-scale simulation, parallel computing, computational neuroscience

## Abstract

Investigating the dynamics and function of large-scale spiking neuronal networks with realistic numbers of synapses is made possible today by state-of-the-art simulation code that scales to the largest contemporary supercomputers. However, simulations that involve electrical interactions, also called gap junctions, besides chemical synapses scale only poorly due to a communication scheme that collects global data on each compute node. In comparison to chemical synapses, gap junctions are far less abundant. To improve scalability we exploit this sparsity by integrating an existing framework for continuous interactions with a recently proposed directed communication scheme for spikes. Using a reference implementation in the NEST simulator we demonstrate excellent scalability of the integrated framework, accelerating large-scale simulations with gap junctions by more than an order of magnitude. This allows, for the first time, the efficient exploration of the interactions of chemical and electrical coupling in large-scale neuronal networks models with natural synapse density distributed across thousands of compute nodes.

## 1. Introduction

Electrical synapses, called gap junctions, are channels in the membranes of two neighboring cells allowing a direct bidirectional exchange of ions and small molecules (e.g., Bennett and Zukin, 2004). Such seemingly primitive forms of communication have been believed to be relevant mainly in invertebrates after the discovery of chemical synapses, which provide more flexible molecular machinery to modulate the interactions between neurons in vertebrates. However, there is evidence that gap junctions are also widespread in the vertebrate nervous system (e.g., Connors and Long, 2004) where they provide an additional information channel between nerve cells. Today, gap junctions are believed to play a prominent role in the development and function of the nervous system. As they do not involve the typical millisecond delays arising from the transformation between electrical and chemical signals at the synaptic cleft, gap junctions surpass chemical synapses in terms of signaling speed which proves useful, e.g., in escape networks (El Manira et al., 1993; Herberholz et al., 2002). Furthermore, by permitting subthreshold communication between adjacent cells, gap junctions allow efficient propagation of activity waves, they facilitate the synchronization (e.g., Bennett and Zukin, 2004; Mancilla et al., 2007; Laing, 2015) or desynchronization of neuronal populations (Vervaeke et al., 2010), and they can enhance the integration of excitatory inputs via coupling of dendritic trees (Vervaeke et al., 2012). Far from being a primitive form of signaling, gap junctions have been shown to support activity-dependent and neuromodulator-dependent plasticity (Pereda et al., 2004, 2013; Pernelle et al., 2018), and they can rectify signals via a different efficacy of transmission in each direction (Furshpan and Potter, 1959). Moreover, dysfunction in gap-junction coupling has been linked to neurological disorders such as epilepsy (e.g., Mas et al., 2004). The connectivity of neurons via gap junctions is fundamentally different from that via chemical synapses. Gap junctions mainly couple inhibitory interneurons of the same type (e.g., Bennett and Zukin, 2004) in a locally restricted area of up to 500 μm (Fukuda, 2007). While each cortical cell has on average several thousands of connections via chemical synapses, gap-junction connections are much sparser, each cell being coupled to about 60 of its neighboring cells (Fukuda, 2007).

It is now assumed that the functions of chemical and electrical coupling in the nervous system are not independent but complementary (Hormuzdi et al., 2004; Pereda, 2014), which emphasizes the need to investigate both types of coupling in computational models. Computational modeling serves as an important tool for scientific discovery by allowing researchers to quickly explore the dynamics and function of mechanistic models that are difficult to handle analytically without severe simplifications. While a plethora of different network models consider the effect of chemical synapses, relatively few models investigate the role of gap junctions on the dynamical, functional, and behavioral level, let alone their role in nervous-system dysfunction. Topics of present studies include: investigation of the effect of gap junctions on the synchronization of neuronal networks (Pfeuty et al., 2003; Holzbecher and Kempter, 2018), demonstration of the emergence of oscillations (Tchumatchenko and Clopath, 2014) and efficient propagation of traveling waves due to subthreshold coupling, and recently also modeling of gap-junction plasticity (Pernelle et al., 2018). The present network models, however, are of relatively small size compared to, for example, the number of neurons just under one square millimeter of cortical surface. To faithfully reproduce network dynamics and predict coarse-grained measures that can be related to neuronal recordings one needs to simulate full-scale models (van Albada et al., 2015). However, large-scale simulations at single neuron resolution and with realistic connection density require simulation technology that can efficiently distribute the workload across many compute nodes.

The efficient distributed simulation of spiking neuronal networks relies on the propagation of neuronal state variables on a time grid with a time interval in the sub-millisecond range (e.g., 0.1 ms). As spiking interactions are typically delayed, they allow for communication of spike data at larger time intervals (e.g., 1 ms) while still maintaining causality (Morrison et al., 2005). This reduction in communication frequency constitutes an important optimization in neuronal network simulation technology. Firstly, it decreases the communication costs and, secondly, the optimization improves cache utilization during neuronal updates since the state variables of the individual neurons are propagated for an entire communication interval with substeps defined by the neuronal update interval. On first assessment, incorporating electrical coupling through gap junctions in such simulations seems to conflict with the reduced communication frequency: In a time-driven simulation scheme, a typical gap junction is modeled via an interaction of the form

(1) $$\begin{array}{l} I_{\text{gap},ij}\left(t\right)=g_{ij}\left(V_{i}\left(t\right)-V_{j}\left(t\right)\right) \end{array}$$

between neuron *i* and *j* (e.g., Pfeuty et al., 2003; Bennett and Zukin, 2004; Mancilla et al., 2007). Computing the current *I*_gap,*ij*_ requires that the membrane potential *V*_*i*_ of neuron *i* is available to neuron *j* at all times *t* (*I*_gap,*ij*_ positive if current flows from *i* to j as in Mancilla et al., 2007), and vice versa. Here, *g*_*ij*_ represents the symmetric, possibly weakly voltage-dependent (e.g., Bennett and Zukin, 2004) coupling strength of the gap junction. Coupling neurons that reside on different compute nodes of a parallel computer via a gap junction thus requires the communication of membrane potential data on the same time scale as neuronal updates. This entails increased communication costs with regard to both latency and bandwidth, thus limiting scalability.

To address this issue, Hahne et al. (2015) introduced a simulation technology for incorporating continuous coupling between neurons in spiking neuronal networks using a numerical interpolation scheme based on wave-form relaxation methods (Lelarasmee et al., 1982). The novel framework meets the requirements of simulations with continuous interactions while still allowing for communication of spikes and continuous data in intervals larger than the neuronal update step. This enables researchers to investigate the interaction between chemical and electrical coupling believed to be important for healthy brain function (Pereda, 2014). However, the framework exhibits suboptimal scaling properties in terms of runtime and memory consumption, both of which increase linearly with the number of MPI processes for a fixed problem size per process. This limits the practical use of the technology for large-scale simulations using several hundreds of compute nodes. The framework was later extended to support rate-based connections (Hahne et al., 2017), the scalability issues, however, remained. Technically gap junctions and rate-based connections require similar capabilities in a simulator: both lead to an instantaneous coupling between two neurons. Hence, we also refer to such connections using the generalizing term “continuous-data connections” in contrast to the event-based coupling via spikes, which we refer to as “spiking connections.”

**Figure 1:**
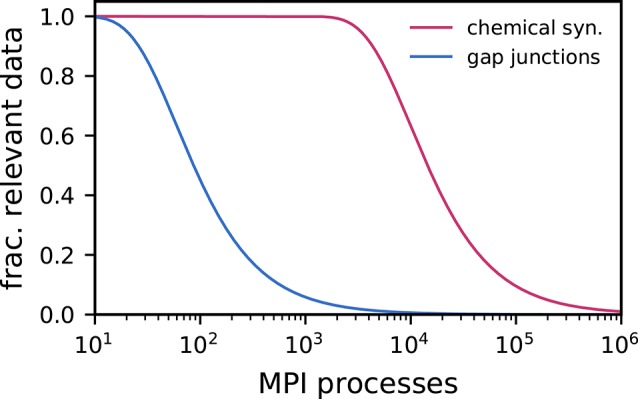
Fraction of activity data relevant for a given MPI process decreases as a result of increasingly sparse connectivity. In weak-scaling scenarios, the fraction of relevant spike data (pink) and gap-junction data (blue) decreases as the number of MPI processes and thus the total network size increases: an ever smaller fraction of the data produced by the network is relevant for the synapses and neurons represented locally on a given MPI process. The effect is more pronounced for gap-junction data than for spike data due to the difference between the typical numbers of gap junctions and chemical synapses per neuron. Here, we assume *N*_*M*_ = 10, 000 neurons per MPI process and either *K* = 10, 000 incoming chemical synapses per neuron (pink) or *K*_gap_ = 60 gap junctions per neuron (blue) respectively. See Supplementary Material for derivation.

Jordan et al. (2018) presented a technology tackling scalability issues for networks with purely chemical coupling exploiting the sparsity of large-scale cortical networks. By introducing a two-tier connection infrastructure, which comprises complementary data structures on both sending and receiving side, allows for directed communication of spikes. This significantly improves the scalability of large-scale spiking neuronal network simulations with regard to runtime and memory usage. Due to the small number of gap junctions per cell (Fukuda, 2007) compared to typical numbers of chemical synapses, the sparsity of gap-junction coupling in large-scale neuronal networks as a function of the number of MPI processes becomes relevant earlier (Figure 1). Once the number of MPI processes exceeds the typical number of outgoing connections per neuron, most of the data collected on each process is not locally required. This indicates that simulations of such networks can significantly benefit from exploiting the extreme sparsity of gap junctions through a directed communication scheme.

In this study, we describe how the framework for continuous interactions is integrated with the two-tier connection infrastructure and the directed communication scheme. We demonstrate significantly improved scalability of network simulations with gap junctions: For networks of about 600, 000 neurons connected via 36 million gap junctions simulation time is reduced by more than a factor of 20 with respect to the previous technology. Therefore, the new technology enables a systematic numerical investigation of the dynamics of large-scale cortical network models with both chemical and electrical coupling.

The remainder of this work is organized as follows: In section 2 we introduce the network model used for comparing the performance of the previous and new framework for continuous interactions, we provide key data of the HPC system employed for scaling experiments, and we introduce the NEST simulator for which we provide a reference implementation. In section 3, we first highlight the differences between the communication of spike data and continuous data, then we present the necessary changes to the two-tier connection infrastructure and the directed communication algorithm that allows for continuous interactions in parallel to spiking interactions, and finally, we compare the performance of the previous and the new simulation technology based on weak-scaling and strong-scaling scenarios. The study concludes by discussing limitations of the current technology and benchmark models, future extensions, and modeling prospects.

The technology described in the present article will be made available to the community with one of the next releases of the open-source simulation software NEST. The conceptual and algorithmic work described here is a module in our long-term collaborative project to provide the technology for neural systems simulations (Gewaltig and Diesmann, 2007).

## 2. Materials and Methods

### 2.1. Benchmark Network Model

We compare memory usage and runtime of the previous technology to the new framework for continuous-data connections using the scalable network model introduced in Hahne et al. (2015), which consists of *N* single-compartment Hodgkin-Huxley neuron models with alpha-shaped postsynaptic currents (in NEST: hh_psc_alpha_gap) connected by gap junctions. As we investigate the scaling properties of continuous coupling, we do not make use of chemical synapses. The NEST implementation of the model uses the Runge-Kutta-Fehlberg solver (gsl_odeiv_step_rkf45) of the GNU Scientific Library with an adaptive step-size control (gsl_odeiv_control_y_new) to advance the state of an individual neuron. The network model exhibits ring topology where each neuron is connected to 60 of its nearest neighbors. Each neuron receives a noise current that, for each simulation time step of duration *h*, is drawn independently from a Gaussian distribution with mean 200 pA and standard deviation 250 pA; note that the resulting variance of the filtered synaptic current thus depends on the time resolution and is ∝ *h* for small time steps. All other neuron-model parameters are as defined in Mancilla et al. (2007). All gap junctions have identical weight *g* = 0.1 nS. The simulation step is *h* = 0.1 ms, and the communication interval is 1 ms. The details of the network model are given in the Supplementary Material.

In all simulations we use the following waveform-relaxation parameters (Hahne et al., 2015): maximal number of iterations is 5, tolerance is 10^−5^, and interpolation order is 3.

### 2.2. Benchmark System: JURECA

For the scaling experiments in section 3.5 we use up to 1664 compute nodes of the JURECA HPC system located at the Jülich Supercomputing Centre (JSC) in Jülich, Germany. Each node is equipped with two Intel Xeon E5-2680 v3 Haswell CPUs and a minimum of 128 GiB of memory. Communication between compute nodes is enabled by a Mellanox EDR InfiniBand high-speed network with non-blocking fat tree topology. For all simulations we used two MPI processes per node and 12 OpenMP threads per process. NEST was compiled with GCC 8.2.0 and ParaStation MPI 5.2.1-1.

### 2.3. NEST Simulator

NEST is an open-source software tool for simulations of large-scale networks of spiking neuron models (Gewaltig and Diesmann, 2007). The NEST Initiative^1^ maintains NEST with the goal of long-term availability. The developer community actively contributes new features, bug fixes, and documentation. NEST is licensed under the GNU General Public License, version 2^2^ and can be freely downloaded from the website of the NEST simulator^3^. While the simulation kernel is implemented in C++, specifications of simulations are performed in interpreted languages: the built-in scripting language SLI or Python (PyNEST; Eppler et al., 2009; Zaytsev and Morrison, 2014). A hybrid programming model allows running a combination of MPI processes for distributed computation and threads for lightweight parallelization within compute nodes (Ippen et al., 2017). NEST scales well throughout an entire range of platforms — from laptops to supercomputers (Jordan et al., 2018), and it supports advanced model components, for example neuromodulated plasticity (Potjans et al., 2010), structural plasticity (Diaz-Pier et al., 2016), coupling between neurons via gap junctions (Hahne et al., 2015), and non-spiking neurons with continuous interactions such as rate-based models (Hahne et al., 2017). The simulation code used for the benchmarks is based on the NEST 2.14 release, with Git SHA ba8aa7e (4g) and 0f8d5b5 (5g), respectively. Source code, simulation and analysis scripts are available as part of Jordan et al. (2020).

## 3. Results

The minimum synaptic transmission delay in spiking neuronal network models allows for a fundamental optimization: For the duration of the minimum delay individual neuron dynamics are decoupled and, hence, can be independently propagated, such that the communication of spikes can take place on a coarser time grid than neuron updates (Morrison et al., 2005). This reduces communication costs in distributed simulations. The coupling of neurons via gap junctions is at odds with such an optimized communication scheme as gap junctions lead to an instantaneous coupling of membrane potentials, seemingly requiring communication intervals comparable with the neuronal update interval. To alleviate this issue, Hahne et al. (2015) made use of an efficient iterative method to interpolate membrane potentials over the duration of the minimum synaptic delay. This method allows MPI communication to again take place on a time grid larger than that of neuron updates, reducing communication costs while maintaining high precision. The framework introduced by Hahne et al. (2015) made use of MPI Allgather (Message Passing Interface Forum, 2009) to communicate continuous-data events, which was an efficient communication scheme for spiking network simulations at the time. The scheme is compatible with the characteristics of small- to medium-scale simulations: As long as the number of MPI processes is not much larger than the number of connections per neuron, each neuron's data is likely required on every process. All data is hence collected on each process and irrelevant data is only discarded after the communication. However, this turns into a suboptimal strategy in regimes where most of the received data is not locally required on each process. This is already the case for a few hundreds of MPI processes in the case of gap junctions (Figure 1). Here, we describe how the two-tier connection infrastructure and the directed communication scheme introduced for spike data in Jordan et al. (2018) is extended to support continuous-data connections such as gap junctions, eliminating the communication of irrelevant data.

### 3.1. Spiking and Continuous-Data Connections Imply Distinct Requirements for Communication

Hahne et al. (2015) presented a unified framework for both spiking and continuous interactions, where the same data structure stores continuous-data connections as well as spiking connections. Moreover, the delivery of continuous-data events to local targets, which takes place after the MPI communication, follows the concept for the delivery of spike data using a source-based address-event representation protocol (AER; Boahen, 2000; Lansner and Diesmann, 2012). Such an incremental development process that extends and reuses existing data structures and algorithms simplifies testing and maintenance of the code base and allows new features to be released more quickly to the community.

**Figure 2:**
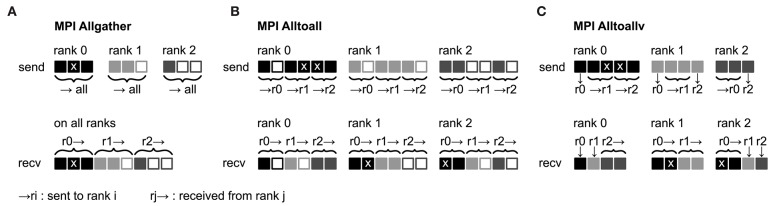
Collective communication of receiver-selective data using MPI Allgather, MPI Alltoall, and MPI Alltoallv. The three panels illustrate send and receive buffers for the example of an MPI communication that involves three ranks. Homogeneous data types are required throughout the entire send and receive buffer. Squares represent single buffer entries. Data sent by rank 0, rank 1, and rank 2 are shown in dark gray, light gray, and medium gray, respectively. To indicate receiver selectivity, we define the data marked with “x” to be required on rank 1 and rank 2 but not on rank 0. **(A)** MPI Allgather: All ranks receive the entire send buffer from all ranks, which can include irrelevant data, for example, the receive buffer on rank 0 contains an “x”-entry with data that is not required. The receive buffer is a concatenation of all send buffers and the receive buffer size hence scales with the total number of ranks taking part in the communication (assuming fixed send buffer sizes). Send buffers of equal size and hence also receive buffers of equal size are required for all ranks, which can entail sending empty buffer entries (unfilled squares). **(B)** MPI Alltoall: Send buffers consist of equally sized sections that are destined for different receiving ranks. This allows each rank to define the data to be transmitted to any particular rank. Each rank has to send identically-sized buffer sections to each rank, which can entail sending empty buffer entries or even entirely empty buffer sections. Rank 2, for example, sends an empty buffer section to rank 1. The size of the receive buffers is identical to the size of the send buffers and independent of the number of ranks participating in the communication. To send specific data to multiple ranks, the sending rank needs to copy the data to the send-buffer sections of all intended receiving ranks, which leads to redundancy in the send buffer; rank 0, for example, sends an “x”-entry to both, rank 1 and 2. **(C)** MPI Alltoallv: Similar to MPI Alltoall, send buffers consist of sections that are destined for different receiving ranks. However, the sections are allowed to differ in size avoiding empty buffer entries. Adapted from Jordan et al. (2018) under CC BY 4.0 (http://creativecommons.org/licenses/by/4.0/).

The unified framework of Hahne et al. (2015) uses a single MPI buffer to communicate spiking and continuous data among MPI processes. Each process maintains a communication buffer for outgoing data, which is communicated to all other processes via MPI Allgather (Figure 2A) such that the global network state is available on each process. While effective for small numbers of processes (Morrison et al., 2005), this type of collective communication is inefficient already for hundreds of MPI processes in simulations with typical numbers of gap junctions (Figure 1). First, large amounts of unnecessary data are communicated if the number of processes exceeds the average number of gap junctions per neuron. Second, during delivery all incoming data in the MPI buffer needs to be read and checked for its relevance to the local neurons. Third, as MPI buffers reflect the global network state, they consume large amounts of memory on each process (see Figure 14 in Hahne et al., 2015).

**Figure 3:**
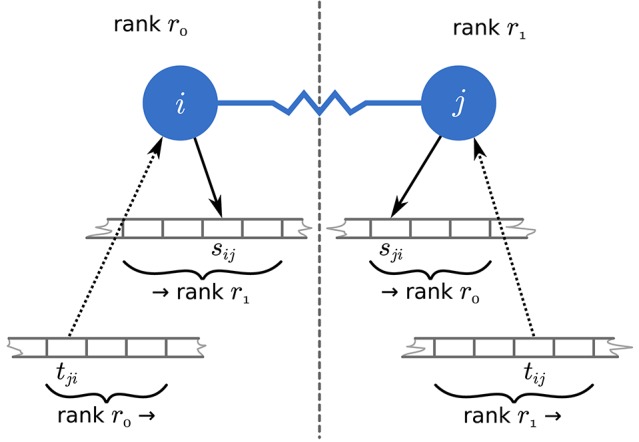
Access to MPI buffers for communication of continuous-data events. Neuron *i* on MPI rank *r*_0_ and neuron *j* on MPI rank *r*_1_ (blue filled circles) are coupled through a gap junction (blue line), which requires them to exchange continuous-data events in every communication step. To this end, each neuron always writes to the same send-buffer position *s*_*ij*_ and *s*_*ji*_, respectively (black solid arrows) and reads from the corresponding receive-buffer positions *t*_*ji*_ and *t*_*ij*_, respectively (black dotted arrows).

To remove these inefficiencies the communication scheme in a neuronal simulator needs to reflect the fundamentally different temporal aspects of spiking, i.e., discrete, and continuous interactions. For spiking interactions, the number of spikes and their senders and receivers differ from one communication step to the next. This requires, for example, temporary buffering of data in spike registers for efficient collocation of MPI buffers in multi-threaded simulations (Morrison et al., 2005; Eppler, 2006). For continuous interactions in networks with a fixed topology however, the amount of data and the corresponding senders and receivers are identical in every communication step. As a consequence there is a unique position in the continuous-data MPI buffer for data from a specific source for a specific target throughout the entire simulation (Figure 3). Here, we exploit this temporal homogeneity to optimize the communication of continuous-data events between neurons. We use separate MPI buffers for spiking and continuous data, where spike data is communicated using MPI Alltoall (Figure 2B) and continuous data is communicated using MPI Alltoallv (Figure 2C). The use of MPI Alltoallv makes most effective use of the MPI buffers and is particularly efficient for the communication of continuous data as we need to compute and communicate the amount of data sent and received among all processes only a single time at the beginning of the simulation. Jordan et al. (2018) presented the algorithms and data structures required for the directed communication in simulations with spiking connections. In the following we describe the necessary changes that support continuous-data connections.

### 3.2. Extension of Two-Tier Connection Infrastructure for Continuous Interactions

To optimize memory consumption and runtime, Jordan et al. (2018) introduced a two-tier connection infrastructure consisting of a postsynaptic part on the process of the target neuron and a presynaptic part on the process of the sending neuron. The postsynaptic part stores the incoming synapses of all process-local neurons and the global identifiers (GIDs) of the corresponding presynaptic neurons. The presynaptic part maintains a list of all targets for every process-local neuron. The data structures allow the use of directed communication methods (MPI Alltoall) to exclusively send spike data from a presynaptic neuron to the processes hosting the corresponding postsynaptic partners. Here, we adapt the two-tier connection infrastructure to continuous-data connections and, hence, the directed communication of continuous-data events.

#### 3.2.1. Postsynaptic Connection Infrastructure

The original postsynaptic part of the connection infrastructure for spiking connections presented in Jordan et al. (2018) consists of two identically structured three-dimensional resizable arrays: the first stores connection objects indexed by incoming spike events using a target-based AER scheme, while the second keeps track of the corresponding presynaptic sources. The latter is required to instantiate the presynaptic part of the connection infrastructure at the beginning of the simulation and to speed up user queries about network connectivity. Both structures are continuously populated as connections are created.

**Figure 4:**
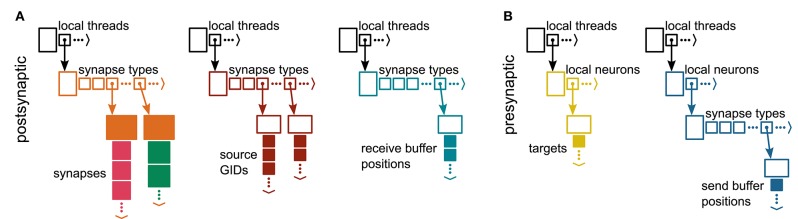
Two-tier connection infrastructure for spiking and continuous-data connections on each MPI process. All components of the connection infrastructure hold data separated by thread using a resizable array of pointers to the thread-local data structures (top, black). **(A)** Postsynaptic side. The receiver side of the connection infrastructure consists of three identically structured parts: the connection table (left), the source table (middle), and the table of receive-buffer positions for continuous-data connections (right). Connection table: Thread-specific resizable arrays store pointers to variable-sized containers for every synapse type. If a synapse type is in use, the corresponding container (orange filled rectangle) stores all thread-local connections of this type in a resizable array; both spiking connections (pink filled squares) and continuous-data connections (green filled squares) are stored. Synapse types can differ in memory consumption per object, indicated by different sizes of the synapse objects. Source table: Source objects (dark red filled squares) are stored in a three-dimensional resizable array, with a one-to-one relation between each source object and the connection object in the same location in the connection table. Sources contain the GIDs of the presynaptic neurons. Table of receive-buffer positions: Thread-specific two-dimensional resizable arrays store receive-buffer positions (turquoise filled squares) for all continuous-data connections, with a one-to-one relation between each buffer position and the continuous-data connection object in the same location in the connection table. **(B)** Presynaptic side. The sender side of the connection infrastructure consists of the target table (left) and the table of send-buffer positions for continuous-data connections (right). Target table: Thread-specific two-dimensional resizable arrays store the target objects (yellow filled squares) for every thread-local neuron. The target objects contain the locations of the targets in terms of the MPI ranks and the locations in the corresponding connection table on the postsynaptic side. Table of send-buffer positions: Thread-specific three-dimensional arrays store the send-buffer positions (blue filled squares) for the outgoing continuous-data connections of every thread-local neuron. Adapted from Jordan et al. (2018) under CC BY 4.0 (http://creativecommons.org/licenses/by/4.0/).

To enable continuous-data connections we reuse the data structures to store the connection objects and the corresponding presynaptic GIDs (Figure 4A). In addition, we introduce another identically structured resizable array that stores for each continuous-data connection the corresponding MPI receive-buffer position where presynaptic data for this connection is read from (see also Figure 3). This additional data structure increases the memory consumption per continuous-data connection but it allows efficient delivery of continuous-data events as described in section 3.4.

#### 3.2.2. Presynaptic Connection Infrastructure

The original presynaptic part of the connection infrastructure for spiking events consists of a three-dimensional resizable array storing the targets of each local neuron. The target information of a sending neuron is used to communicate spikes only to the processes that host the postsynaptic targets. Essentially, the target information comprises the locations of target synapses in the postsynaptic data structure (see Jordan et al., 2018 for details).

To enable directed communication of continuous-data events, we extend the presynaptic connection infrastructure with a four-dimensional resizable array (Figure 4B). For each process-local neuron with outgoing continuous-data connections the new structure stores MPI send buffer positions separated by their synapse type. The stored positions allow the sending neurons to directly write events to the correct locations in the MPI send buffer, allowing efficient filling of the buffer (see section 3.4).

### 3.3. Construction of Extended Connection Infrastructure

When creating continuous-data connections, only the data structures storing the actual connection objects and the corresponding presynaptic GIDs are filled. Whereas, the data structures storing the targets and the MPI buffer positions are constructed at the beginning of the simulation or upon request by the user. Their construction is performed in multiple subsequent steps. First, all connections in the postsynaptic infrastructure are sorted by the source neuron GIDs for each synapse type. This is an optional step and part of an optimization for small-scale to medium-scale neuronal network simulations (see Jordan et al., 2018 for details). Then, receive buffer positions are computed for each continuous-data connection as follows: (i) for every continuous-data connection retrieve the source GID and the synapse type, (ii) create a set of unique pairs of source GID and synapse type across all threads, (iii) iterate over this set to compute the relative receive buffer positions for each pair according to the MPI rank of the source neuron, while keeping track of the total amount of data to be expected from every MPI rank, (iv) translate the relative receive buffer positions for every continuous-data connection into absolute positions, and finally, (v) store the obtained absolute positions in the three-dimensional array of receive buffer positions. Note that in (iii) the MPI rank of the source neuron can be obtained from the GID of the neuron due to the round-robin distribution of neurons across MPI processes and threads.

Having thus completed the postsynaptic part of the connection infrastructure, the presynaptic part can be constructed. The construction of the presynaptic part of the infrastructure for continuous-data connections is performed together with the construction of the presynaptic infrastructure for spiking connections (cf. Jordan et al., 2018, section 3.1.3). However, while an entry in the presynaptic structure for spiking connections encodes the location of a connection in the postsynaptic connection infrastructure on a specific MPI rank, the entry for a continuous-data connection is an index in the MPI send buffer. The send buffer positions are computed from the receive buffer positions received from every MPI rank, taking into account the rank of the target neuron and the total amount of data sent to each rank. During construction of the presynaptic infrastructure we make sure that every send buffer position only appears once in the target list, to avoid redundant writing to the MPI buffer. Note that after gathering this information from each rank one can easily compute the exact amount of data that will be sent to each rank in every communication step, a prerequisite for using MPI Alltoallv.

### 3.4. Communication of Continuous Data

Communicating continuous-data events using the new two-tier connection infrastructure is straightforward. In contrast to the previous technology (Hahne et al., 2015), MPI buffers for spiking events and continuous-data events are separate and communicated independently from each other. When a neuron generates a continuous-data event, it retrieves the send buffer positions from the corresponding data structure for all synapse types that are able to handle the generated event (in NEST: e.g., GapEvent or RateEvent). The neuron then writes the data it needs to communicate to the corresponding position in the MPI send buffer. At the end of the communication interval the buffers are communicated across all ranks using MPI Alltoallv. After the communication is completed, the delivery of continuous-data events is performed by iterating over all entries in the data structure storing MPI receive buffer positions. For all continuous-data connections the data is retrieved from the corresponding position in the MPI receive buffer and then delivered via the synapse objects to the target neurons. In contrast to the previous technology the new communication scheme does not require temporary buffering of continuous data and supports fully thread-parallel sending and delivery of events.

### 3.5. Performance of New Framework for Continuous Interactions

Our analysis of the performance of the new simulation technology for continuous interactions, henceforth referred to as “5g,” and the comparison to the previous technology (Hahne et al., 2015, “4g”) are based on reference implementations in the NEST simulator (section 2.3). All scaling experiments are performed on the JURECA supercomputer (section 2.2) using the example network described in section 2.1. As in Jordan et al. (2018), we distinguish between “build time,” “init time,” and “sim time.” The build time accounts for network construction, not including the construction of the presynaptic data structure and the resizable arrays storing MPI buffer positions, which is taken into account in the init time, a separately timed initial simulation period of 10 ms. The sim time accounts for the actual simulation of 0.5 s of biological time.

#### 3.5.1. Weak Scaling

**Figure 5:**
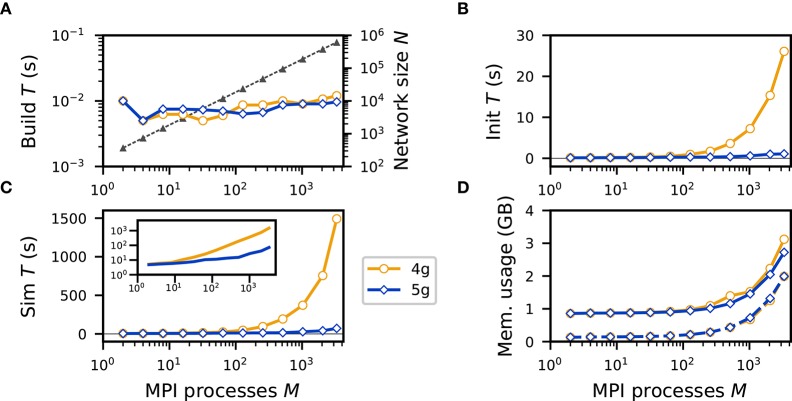
Weak scaling of neuronal network simulation with gap junctions on a petascale computer. Runtime and memory usage per compute node for an increasing number of MPI processes in logarithmic representation *M* ∈ {2; 4; 8; 16; 32; 64; 128; 256; 512; 1024; 2048; 3328} with two MPI processes per compute node and *T* = 12 threads per MPI process on JURECA. Each compute node hosts *N*_*M*_ = 185 neurons with *K* = 60 gap junctions per neuron. Network dynamics simulated for 0.5 s of biological time. Color code in all panels (legend): 4g (orange, open circles), 5g (blue, open diamonds). **(A)** Build time (logarithmic). Gray triangles and dashed line show the total network size *N* (right vertical axis, logarithmic). **(B)** Init time. **(C)** Sim time. Inset shows same data with logarithmic vertical axis. **(D)** Cumulative memory usage for a single MPI process after NEST startup (dashed curves) and after simulation (solid curves).

In a weak-scaling scenario the problem size per process is fixed, here, the number of neurons and connections per process, while the number of processes increases. This leads to an increase of network size with the number of processes. As the compute resources are scaled in proportion, one expects constant runtime and memory consumption for a perfectly scalable application. Here, we simulate *NM* = 185 neurons per MPI process and *K*_gap_ = 60 gap junctions per neuron (see section 2.1). We consider a structured network with ring topology: each neuron is connected to a fixed number of its nearest neighbors, where neighborhood between neurons is defined according to their order of creation, indicated by their respective GIDs. The build time is identical for both 4g and 5g and independent of the number of MPI processes (Figure 5A), as the generation of connections is fully parallel. The init time is similar between the previous (4g) and the new technology (5g) up to about 100 MPI processes but then significantly increases for 4g (Figure 5B). Due to the small number of connections per neuron the construction of the presynaptic connection infrastructure takes a negligible amount of time for 5g. Hence, despite its short duration of 10 ms, the init time is dominated by the propagation of network dynamics, which has much better scaling behavior for 5g than for 4g. This is also reflected in the sim time: already at 512 processes the runtime of simulations with 5g is more than an order of magnitude smaller than with 4g (Figure 5C, 15.17 s vs. 193.00 s). The poor scalability of simulation with 4g renders large-scale modeling studies inefficient, whereas for 5g the simulation duration only increases by a factor of about 15 from 2 processes to 3328 processes, while network size grows by a factor of 1664 from 370 to 615, 680 neurons. The largest network simulated in this study with the new technology comprises 615, 680 neurons connected by ~36.9 million gap junctions and requires 71.90 s of wallclock time for 0.5 s of biological time. The memory consumption after simulation is constant for 4g and 5g up to about 128 MPI processes. Then the memory usage of 4g increases linearly with the number of processes, mainly due to the increase of MPI buffer sizes (Hahne et al., 2015) in addition to the increased memory usage by the MPI library (Figure 5D). For 5g the memory consumption also increases due to an increasing overhead of the MPI library as measured directly after startup of the NEST kernel before any neurons or connections are created (Figure 5D). The smallest simulations exhibit only marginal runtime and memory differences between the two implementations confirming that the changes to the connection infrastructure and communication algorithms described in section 3.2 do not impair typical small-scale simulations on laptops and workstations.

#### 3.5.2. Strong Scaling

**Figure 6:**
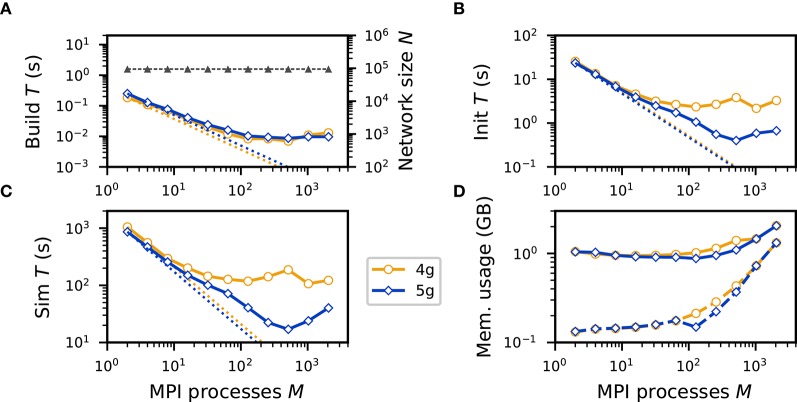
Strong scaling of neuronal network simulation with gap junctions on a petascale computer. Runtime and memory usage per compute node for an increasing number of MPI processes in logarithmic representation *M* ∈ {2; 4; 8; 16; 32; 64; 128; 256; 512; 1024; 2048; 3328} with two MPI processes per compute node and *T* = 12 threads per MPI process on JURECA. *N* = 94, 720 neurons with *K* = 60 gap junctions per neuron. Network dynamics simulated for 0.5 s of biological time. Color code and marker styles as in Figure 5; logarithmic vertical axes. For reference, straight dotted lines indicate perfect scaling. **(A)** Build time (logarithmic). Gray triangles and dashed line show the total network size N (right vertical axis, logarithmic). **(B)** Init time. **(C)** Sim time (logarithmic). **(D)** Cumulative memory usage for a single MPI process after NEST startup (dashed curves) and after simulation (solid curves).

In a strong scaling scenario the problem size is fixed, here the total number of neurons and connections, while the number of processes increases. Perfect scaling would result in a decrease in runtime inversely proportional to the number of processes. We consider a network of *N* = 94, 720 neurons, which reflects the typical number of neurons in a cortical column, and *K*_gap_ = 60 gap junctions per neuron. As in the weak-scaling scenario, we consider a ring-network topology in which each neuron is connected to its 60 nearest neighbors. The build time decreases with an increasing number of MPI processes, reflecting the parallel nature of the connection algorithm (Figure 6A). However, as ever fewer neurons and connections are represented on each process, serial overhead eventually dominates the build time, such that no significant decrease is observed beyond 128 processes. Due to the small total number of connections in the network, the init time (Figure 6B) is mainly dominated by the propagation of network dynamics and hence exhibits similar scaling behavior as the sim time. The sim time for 4g decreases up to 128 MPI processes, but then becomes stagnant (Figure 6C). In contrast, the sim time for 5g decreases further up to 512 MPI processes. The directed communication via MPI Alltoallv hence has significant benefits also in a strong scaling scenario. For the largest number of processes considered here a network with *N* = 94, 720 neurons coupled by ~5.7 million gap junctions is simulated for 0.5 s of biological time in 17.01 s, making comprehensive investigations of cortical microcircuits with a natural density of gap junctions practically feasible. As in the strong-scaling scenario the network is distributed over an increasing number of processes, one expects memory usage after simulation to decrease with an increasing number of processes. However, we neither observe a decrease for 4g nor for 5g (Figure 6D). This is due to an increased memory usage of the MPI library, which overshadows the decreased memory usage of the simulation (Figure 6D).

## 4. Discussion

Experimental evidence suggests that besides the signal transmission between neurons through chemical synapses, which prevail in vertebrate brains, the less abundant electrical synapses play an important role in neuronal network dynamics and function (Hormuzdi et al., 2004; Pereda, 2014). Interactions through electrical synapses, also called gap junctions, occur locally between neighboring neurons and they mediate direct and continuous reciprocal influence between the membrane potentials of the connected cells. This makes gap-junction interactions fundamentally different from interactions via chemical synapses, which can act remotely through neuronal axons and typically involve signal-transmission delays ranging from a few hundred microseconds to a few milliseconds. Despite the experimental evidence for gap junctions affecting the dynamics of neuronal systems, investigations of networks with both spiking and gap-junction interactions using computational models have been limited to relatively small network sizes. Due to the complex dynamics of chemically and electrically coupled networks, progress in this area of research relies on the availability of appropriate simulation technology that allows for efficient handling of both types of interactions.

An initial hurdle on the way to scalable simulation technology for large-scale spiking neuronal networks with continuous interactions was taken by Hahne et al. (2015). The authors make use of the wave-form relaxation methods of Lelarasmee et al. (1982) in order to enable continuous-data interactions with high precision in globally time-driven simulations where the communication had been optimized taking into account the delays of spiking connections. However, due to the underlying collective communication scheme that performs a complete exchange of data among processes (MPI Allgather), scalability of the framework is limited. In a strong scaling scenario of a network of almost 100, 000 neurons and 6 million gap junctions, the absolute runtime for simulations of 0.5 s of biological time does not decrease below 105 s (Figure 6C) achieving a minimum real-time factor of ~210. This renders a comprehensive investigation of such networks impracticable.

The ratio between the number of connections per neuron and the number of MPI processes used to perform the simulation determines whether a specific communication scheme is efficient. When considering networks with only spiking connections and assuming a typical connection density of, e.g., 10, 000, connections per neuron (Stepanyants et al., 2009), a collective connectivity scheme that transfers all data from each process to all other processes (e.g., MPI Allgather) still transfers mostly relevant data for simulations on up to a few thousand processes. For more processes, the situation changes however: If the number of processes exceeds the number of connections per neuron, a significant fraction of the communicated data is irrelevant, rendering the communication scheme inefficient (Figure 1). In simulations with gap-junction coupling with a typical number of 60 connections per neuron (Fukuda, 2007) such a transition occurs far earlier in a regime below 100 processes.

Jordan et al. (2018) address this issue of increasing sparsity for simulations with purely spiking interactions. In order to extend the scalability of simulation technology for spiking neuronal networks, preparing for the exascale generation of supercomputers, they replace the MPI Allgather communication scheme used to exchange spike data among processes with a collective communication scheme that allows each process to send different data to different processes (MPI Alltoall). To this end, they introduce a two-tier connection infrastructure, which consists of a postsynaptic part as well as a presynaptic part providing information about where to send the spike data on each MPI process. They demonstrate that in benchmark network simulations the new infrastructure and communication scheme are scalable with respect to runtime and memory usage, with significant benefits already on contemporary supercomputers, i.e., in the regime of thousands and tens of thousands of processes.

In this manuscript, we present a highly scalable technology for simulations of large-scale neuronal networks combining spiking and continuous-data interactions. We incorporate the framework for continuous interactions introduced by Hahne et al. (2015) with the extremely scalable connection infrastructure and directed collective communication scheme for spiking interactions presented in Jordan et al. (2018). As the typical number of gap junctions is two orders of magnitude lower than the typical number of spiking connections, we observe significant reductions in runtime with respect to the previous technology (Hahne et al., 2015) already for a few hundred processes, without sacrificing numerical precision.

Using reference implementations of the previous technology (4g) and the new technology (5g) in the NEST simulator, we compare the performance using a benchmark network model with only gap-junction coupling (see section 2.1). The time required for network construction and the memory usage are similar for 4g and 5g (Figures 5A,D, 6A,D). We observe almost perfect scaling with respect to network-construction time. Due to the increased memory usage by the MPI library simulations show poor scalability in this respect but did not reach a critical range for the benchmark network simulated here. Considering the time required to propagate the dynamical state of the network, scalability significantly improves. For a network of about 600, 000 neurons connected via 36 million gap junctions simulation time is reduced by more than a factor of 20 (Figure 5C). For a network of almost 100, 000 neurons and 6 million gap junctions, the minimum runtime for simulations of 0.5 s of biological time reduces to about 17 s (Figure 6C) achieving a minimum real-time factor of ~34. This demonstrates that comprehensive investigations of large-scale spiking neuronal networks with gap junctions are feasible with the new technology.

In case of high computational workload or serial overhead caused by other software components, a poorly scalable communication scheme may not be as exposed as it is in the scaling scenarios shown here. However, communication overhead, in particular overhead of a collective communication scheme that increases with the number of participating MPI processes, ultimately defines a hard lower limit to the runtime that an application can achieve provided that all other bottlenecks can be eliminated. By replacing the MPI Allgather scheme with an MPI Alltoallv scheme supporting variable buffer sizes, the limit to scalability has shifted toward larger numbers of MPI processes in weak-scaling scenarios, and simultaneously also toward lower minimum runtimes in strong-scaling scenarios.

A fundamental strategy in simulation technology for spiking neuronal networks is the round-robin distribution of neurons across MPI processes in order to achieve similar workload on all processes. Consequently, simulations require a collective communication scheme as each MPI process most likely has to communicate with each other process at some point during the simulation (Jordan et al., 2018). This is even more likely if each process can host many neurons, which is the case for simulation technology for point-neuron models, i.e. the technology investigated here. In simulators for networks of many-compartment neurons, such as NEURON (Carnevale and Hines, 2006) and Arbor (Akar et al., 2019) the problem is less pronounced as the investigated networks are typically of smaller size while single neurons are represented at a great level of detail. This typically results in high per-process workload while less data needs to be communicated among MPI processes, such that the overhead caused by communication is less noticeable. The Neural Tissue Simulator (Kozloski and Wagner, 2011) also specializes in detailed representation of neurons but uses a different approach with respect to the distribution of workload, taking into account the three-dimensional organization of neuronal tissue by placing adjacent compartments on the same compute node or on an adjacent node, thus allowing for local communication between MPI processes. Such a distribution strategy seems particularly beneficial for simulations with gap junctions due to their locality.

In the benchmark network model used here, the neuronal dynamics is propagated using a numerical solver with adaptive step size causing a high per-neuron workload as compared to typical benchmark network models with current-based leaky integrate-and-fire (LIF) model neurons, which allow for exact integration (Rotter and Diesmann, 1999). In contrast to integrate-and-fire dynamics, the neuron model describes the shape of the action potential. While for chemical synapses only the time of the spike is relevant, the interaction through electrical synapses is driven by the difference in the voltage excursions of the two cells. A meaningful test of a simulation framework for networks with gap junctions therefore requires a neuron model with an explicit representation of the action potential time course. However, due to the increased computational load of this neuron model, in our study the number of neurons per process is relatively low, which entails lower memory usage and less data to be communicated among processes with respect to typical benchmark networks using simpler neuron models (e.g., Kunkel et al., 2012; Jordan et al., 2018). A higher relative contribution of computation compared to the need of communication trivially leads to better scaling (van Albada et al., 2014). While a detailed account of the contributions of different simulation phases to the total runtime is not within the scope of this work, we speculate that communication still is the limiting factor in the scaling experiments shown here. Therefore, we expect that strong-scaling scenarios of a network of current-based LIF neurons with the same number of neurons and connectivity as the benchmark network model will achieve a similar minimum runtime but already at fewer numbers of processes. This advance over previous simulation technology practically enables researchers to investigate the interaction between chemical and electrical coupling in network models of the size of the cortical microcircuit with natural neuron and connection densities (e.g., Potjans and Diesmann, 2014).

The here assumed, experimentally obtained estimate of 60 gap junctions per neuron (Fukuda, 2007) might not apply in general across all neuronal systems of interest. If the modeled number of gap junctions per cell is higher, this leads to a corresponding increase of MPI buffer size required to communicate the continuous data events. The frequency of collective communication calls would however remain unchanged. A significant increase in buffer size entails an increase in communication time. However, even an increase in the number of gap junctions by two orders of magnitude would not result in a situation where each MPI process requires all data from all other processes, such that an MPI Alltoallv scheme with varying buffer sizes would still be more efficient than an MPI Allgather scheme. The technology presented here should hence be tolerant to such challenges.

The benchmark network model used here assumes ring-topology by coupling each neuron to its nearest neighbors. This topology reflects the locality of gap junctions in neuronal tissue. Due to the round-robin distribution of neurons and a collective communication scheme in which each MPI process communicates with all other processes, we expect network topology to only marginally influence the communication times. A comprehensive analysis of the performance of the communication scheme for skewed distributions of the per-receiver send buffer fractions caused by different network topologies is, however, not within the scope of this study. For the efficient construction of randomly connected networks with gap junctions in NEST, a parallel implementation of a connection algorithm for bidirectional couplings is still missing (Hahne et al., 2015).

We employed a standard single-compartment Hodgkin-Huxley neuron model with passive gap-junction coupling. In order to explore the variety of effects on electrical coupling in neuronal tissue, new neuron models that support subthreshold coupling and more complex gap-junction connections need to be established in future work. Promising candidates include voltage-gated gap junctions, various types of activity-dependent or neuromodulator dependent gap-junction plasticity models or even more complex heterogeneous synapses that involve both chemical and electrical interactions (Pereda, 2014).

The technology we presented here is implemented using separate communication buffers for continuous data and spike data. Maintaining separate buffers is convenient due to the different strategies for accessing them, and due to simulations with gap junctions possibly requiring several communication rounds in order to iteratively obtain a desired target precision of membrane potentials. However, if communication latency is a bottleneck, it is possible to combine the communication of both types of events into a single MPI buffer by merging the buffer part for continuous data with the respective buffer parts for spike data. The efficiency gain of such an approach needs to be carefully evaluated.

In the current implementation, the construction of the presynaptic part of the connection infrastructure for continuous-data connections relies on the same algorithms as the construction of the presynaptic data structures for spiking connections. This implementation choice is mainly to control complexity, however it entails a penalty in runtime as redundant information is sent to the presynaptic side: If a source neuron has multiple targets on a postsynaptic process distributed across several threads, each of the threads communicates the same data to the sending process when constructing the presynaptic infrastructure; the redundant information is only discarded after arriving on the presynaptic process. The construction of the presynaptic data structures takes place only once at the beginning of the simulation. Nevertheless, for increasing numbers of threads per compute node, the redundancy in the communicated data could impair performance. Further analysis is required to check whether the decrease in runtime warrants an increase in implementation complexity.

Due to their prevalence in the vertebrate nervous system and technological challenges, many computational studies focus on chemical synapses and their role in neuronal network function and dysfunction. The technology presented in this article provides researchers with unprecedented possibilities to investigate the interaction of chemical and electrical coupling in large-scale neuronal networks with realistic numbers of synapses. We hope that our work encourages further research into the complementary function of chemical and electrical synapses in the nervous system.

## Data Availability Statement

The datasets generated for this study can be found in Jordan et al., 2020.

## Author Contributions

All authors listed have made a substantial, direct and intellectual contribution to the work, and approved it for publication.

## Conflict of Interest

The authors declare that the research was conducted in the absence of any commercial or financial relationships that could be construed as a potential conflict of interest.

## Footnotes

^1^https://nest-initiative.org/

^2^https://www.gnu.org/licenses/old-licenses/gpl-2.0.html

^3^https://www.nest-simulator.org/

## References

[B1] Akar N. A. Cumming B. Karakasis V. Küsters A. Klijn W. Peyser A. (2019). Arbor–A morphologically-detailed neural network simulation library for contemporary high-performance computing architectures, in 2019 27th Euromicro International Conference on Parallel, Distributed and Network-Based Processing (PDP) (Pavia: IEEE), 274–282.

[B2] Bennett M. V. Zukin R. S. (2004). Electrical coupling and neuronal synchronization in the mammalian brain. Neuron 41, 495–511. 10.1016/S0896-6273(04)00043-1 14980200

[B3] Boahen K. A. (2000). Point-to-point connectivity between neuromorphic chips using address events. IEEE Trans. Circuits II 47, 416–434. 10.1109/82.842110

[B4] Carnevale N. T. Hines M. L. (2006). The NEURON Book. Cambridge: Cambridge University Press.

[B5] Connors B. W. Long M. A. (2004). Electrical synapses in the mammalian brain. Annu. Rev. Neurosci. 27, 393–418. 10.1146/annurev.neuro.26.041002.131128 15217338

[B6] Diaz-Pier S. Naveau M. Butz-Ostendorf M. Morrison A. (2016). Automatic generation of connectivity for large-scale neuronal network models through structural plasticity. Front. Neuroanat. 10:57. 10.3389/fnana.2016.00057 27303272 PMC4880596

[B7] El Manira A. Cattaert D. Wallen P. DiCaprio R. Clarac F. (1993). Electrical coupling of mechanoreceptor afferents in the crayfish: a possible mechanism for enhancement of sensory signal transmission. J. Neurophysiol. 69, 2248–2251. 10.1152/jn.1993.69.6.2248 8394415

[B8] Eppler J. M. (2006). A multithreaded and distributed system for the simulation of large biological neural networks (Master's thesis). Freiburg: Albert Ludwig University Freiburg.

[B9] Eppler J. M. Helias M. Muller E. Diesmann M. Gewaltig M. (2009). PyNEST: a convenient interface to the NEST simulator. Front. Neuroinform. 2:12. 10.3389/neuro.11.012.2008 19198667 PMC2636900

[B10] Fukuda T. (2007). Structural organization of the gap junction network in the cerebral cortex. Neuroscientist 13, 199–207. 10.1177/1073858406296760 17519363

[B11] Furshpan E. Potter D. (1959). Transmission at the giant motor synapses of the crayfish. J. Physiol. 145, 289–325. 10.1113/jphysiol.1959.sp006143 13642302 PMC1356828

[B12] Gewaltig M.-O. Diesmann M. (2007). NEST (NEural Simulation Tool). Scholarpedia 2:1430 10.4249/scholarpedia.1430

[B13] Hahne J. Dahmen D. Schuecker J. Frommer A. Bolten M. Helias M.. (2017). Integration of continuous-time dynamics in a spiking neural network simulator. Front. Neuroinform. 11:34. 10.3389/fninf.2017.00034 28596730 PMC5442232

[B14] Hahne J. Helias M. Kunkel S. Igarashi J. Bolten M. Frommer A.. (2015). A unified framework for spiking and gap-junction interactions in distributed neuronal network simulations. Front. Neuroinform. 9:22. 10.3389/fninf.2015.00022 26441628 PMC4563270

[B15] Herberholz J. Antonsen B. L. Edwards D. H. (2002). A lateral excitatory network in the escape circuit of crayfish. J. Neurosci. 22, 9078–9085. 10.1523/JNEUROSCI.22-20-09078.2002 12388615 PMC6757705

[B16] Holzbecher A. Kempter R. (2018). Interneuronal gap junctions increase synchrony and robustness of hippocampal ripple oscillations. Euro. J. Neurosci. 48, 3446–3465. 10.1111/ejn.14267 30414336

[B17] Hormuzdi S. Filippov M. Mitropoulou G. Monyer H. Bruzzone R. (2004). Electrical synapses: a dynamic signaling system that shapes the activity of neuronal networks. Biochim. Biophys. Acta 1662, 113–137. 10.1016/j.bbamem.2003.10.023 15033583

[B18] Ippen T. Eppler J. M. Plesser H. E. Diesmann M. (2017). Constructing neuronal network models in massively parallel environments. Front. Neuroinform. 11:30. 10.3389/fninf.2017.00030 28559808 PMC5432669

[B19] Jordan J. Helias M. Diesmann M. Kunkel S. (2020). NEST 5g Gap Manuscript Scripts, Data, and Sources (Version v1.0.0). Zenodo 10.5281/zenodo.3715041

[B20] Jordan J. Ippen T. Helias M. Kitayama I. Sato M. Igarashi J.. (2018). Extremely scalable spiking neuronal network simulation code: from laptops to exascale computers. Front. Neuroinform. 12:2. 10.3389/fninf.2018.00002 29503613 PMC5820465

[B21] Kozloski J. Wagner J. (2011). An ultrascalable solution to large-scale neural tissue simulation. Front. Neuroinform. 5:15. 10.3389/fninf.2011.00015 21954383 PMC3175572

[B22] Kunkel S. Potjans T. C. Eppler J. M. Plesser H. E. Morrison A. Diesmann M. (2012). Meeting the memory challenges of brain-scale simulation. Front. Neuroinform. 5:35. 10.3389/fninf.2011.00035 22291636 PMC3264885

[B23] Laing C. R. (2015). Exact neural fields incorporating gap junctions. SIAM J. Appl. Math. 14, 1899–1929. 10.1137/15M1011287

[B24] Lansner A. Diesmann M. (2012). Chapter 10: Virtues, pitfalls, and methodology of neuronal network modeling and simulations on supercomputers, in Computational Systems Neurobiology, ed Novére N. L. (Dordrecht: Springer), 283–315.

[B25] Lelarasmee E. Ruehli A. E. Sangiovanni-Vincentelli A. (1982). The waveform relaxation method for time-domain analysis of large scale integrated circuits. IEEE Trans. Comput. Aid Design Integr. Circuits Syst. 1, 131–145. 10.1109/TCAD.1982.1270004

[B26] Mancilla J. G. Lewis T. J. Pinto D. J. Rinzel J. Connors B. W. (2007). Synchronization of electrically coupled pairs of inhibitory interneurons in neocortex. J. Neurosci. 27, 2058–2073. 10.1523/JNEUROSCI.2715-06.2007 17314301 PMC6673558

[B27] Mas C. Taske N. Deutsch S. Guipponi M. Thomas P. Covanis A.. (2004). Association of the connexin36 gene with juvenile myoclonic epilepsy. J. Med. Genet. 41:e93. 10.1136/jmg.2003.017954 15235036 PMC1735851

[B28] Message Passing Interface Forum (2009). MPI: A Message-Passing Interface Standard, version 2.2. Technical report, Knoxville, TN.

[B29] Morrison A. Mehring C. Geisel T. Aertsen A. Diesmann M. (2005). Advancing the boundaries of high connectivity network simulation with distributed computing. Neural Comput. 17, 1776–1801. 10.1162/0899766054026648 15969917

[B30] Pereda A. E. (2014). Electrical synapses and their functional interactions with chemical synapses. Nat. Rev. Neurosci. 15, 250–263. 10.1038/nrn3708 24619342 PMC4091911

[B31] Pereda A. E. Curti S. Hoge G. Cachope R. Flores C. E. Rash J. E. (2013). Gap junction-mediated electrical transmission: regulatory mechanisms and plasticity. Biochim Biophys Acta 1828, 134–146. 10.1016/j.bbamem.2012.05.026 22659675 PMC3437247

[B32] Pereda A. E. Rash J. E. Nagy J. I. Bennett M. V. (2004). Dynamics of electrical transmission at club endings on the mauthner cells. Brain Res. Rev. 47, 227–244. 10.1016/j.brainresrev.2004.06.010 15572174

[B33] Pernelle G. Nicola W. Clopath C. (2018). Gap junction plasticity as a mechanism to regulate network-wide oscillations. PLoS Comput. Biol. 14:e1006025. 10.1371/journal.pcbi.1006025 29529034 PMC5864095

[B34] Pfeuty B. Mato G. Golomb D. Hansel D. (2003). Electrical synapses and synchrony: the role of intrinsic currents. J. Neurosci. 23, 6280–6294. 10.1523/JNEUROSCI.23-15-06280.2003 12867513 PMC6740557

[B35] Potjans T. C. Diesmann M. (2014). The cell-type specific cortical microcircuit: relating structure and activity in a full-scale spiking network model. Cereb. Cortex 24, 785–806. 10.1093/cercor/bhs358 23203991 PMC3920768

[B36] Potjans W. Morrison A. Diesmann M. (2010). Enabling functional neural circuit simulations with distributed computing of neuromodulated plasticity. Front. Comput. Neurosci. 4:141. 10.3389/fncom.2010.00141 21151370 PMC2996144

[B37] Rotter S. Diesmann M. (1999). Exact digital simulation of time-invariant linear systems with applications to neuronal modeling. Biol. Cybern. 81, 381–402. 10.1007/s004220050570 10592015

[B38] Stepanyants A. Martinez L. M. Ferecskó A. S. Kisvárday Z. F. (2009). The fractions of short- and long-range connections in the visual cortex. Proc. Natl. Acad. Sci. U.S.A 106, 3555–3560. 10.1073/pnas.0810390106 19221032 PMC2651285

[B39] Tchumatchenko T. Clopath C. (2014). Oscillations emerging from noise-driven steady state in networks with electrical synapses and subthreshold resonance. Nat. Commun. 5:5512. 10.1038/ncomms6512 25405458 PMC4243246

[B40] van Albada S. J. Helias M. Diesmann M. (2015). Scalability of asynchronous networks is limited by one-to-one mapping between effective connectivity and correlations. PLOS Comput. Biol. 11:e1004490. 10.1371/journal.pcbi.1004490 26325661 PMC4556689

[B41] van Albada S. J. Kunkel S. Morrison A. Diesmann M. (2014). Integrating brain structure and dynamics on supercomputers, in Brain-Inspired Computing, eds Grandinetti L. Lippert T. Petkov N. (Cham: Springer), 22–32.

[B42] Vervaeke K. Lőrincz A. Gleeson P. Farinella M. Nusser Z. Silver R. A. (2010). Rapid desynchronization of an electrically coupled interneuron network with sparse excitatory synaptic input. Neuron 67, 435–451. 10.1016/j.neuron.2010.06.028 20696381 PMC2954316

[B43] Vervaeke K. Lőrincz A. Nusser Z. Silver R. A. (2012). Gap junctions compensate for sublinear dendritic integration in an inhibitory network. Science 335, 1624–1628. 10.1126/science.1215101 22403180 PMC3587282

[B44] Zaytsev Y. V. Morrison A. (2014). CyNEST: a maintainable Cython-based interface for the NEST simulator. Front. Neuroinform. 8:23. 10.3389/fninf.2014.00023 24672470 PMC3953856

