# Supplementary material for: Efficient Communication in Distributed Simulations of Spiking Neuronal Networks With Gap Junctions
Source: Front Neuroinform. 2020 May 5;14:12. doi: 10.3389/fninf.2020.00012 (PMC7214808; doi:10.3389/fninf.2020.00012)
Supplement: Supplementary file 1 [file Data_Sheet_1.PDF]

Appendix for: "Efficient communication in distributed simulations of spiking neuronal networks with gap junctions"

Jakob Jordan [1,2], Moritz Helias [2,3], Markus Diesmann [2,4,3] and Susanne Kunkel [5]

January 17, 2020

[1] Department of Physiology, University of Bern, Bern, Switzerland
[2] Institute of Neuroscience and Medicine (INM-6) and Institute for Advanced Simulation (IAS-6) and JARA Institute Brain Structure Function Relationship (INM-10), Jülich Research Centre, Jülich, Germany
[3] Department of Physics, Faculty 1, RWTH Aachen University, Aachen, Germany
[4] Department of Psychiatry, Psychotherapy and Psychosomatics, Medical Faculty, RWTH Aachen University, Aachen, Germany
[5] Faculty of Science and Technology, Norwegian University of Life Sciences, Ås, Norway

1 Appendix

1.1 Fraction of relevant data

We define the fraction of relevant data per process as N^+d^+/Nd, where N denotes the total number of neurons, and N^+ denotes the expected number of presynaptic neurons that is relevant for the synapses represented on a particular MPI process and thus their postsynaptic neurons. The average amount of data (spike data or continuous data) produced per neuron during one communication interval is denoted by d considering all neurons or d^+ considering only the relevant neurons. Assuming homogeneous connectivity, the average amount of data produced by each relevant neuron is identical to the average amount per neuron in the network, i.e. $d^+ = d$. The fraction of relevant data per

process is hence just the fraction of relevant neurons N^+/N. Denoting by p_+ the probability that a specific neuron is connected to at least one of the process-local neurons the expected number of relevant neurons is given by $N^+ = p_+ N$. The fraction of relevant data is then just given by the probability p_+. This probability can be written as $p_+ = 1 - p_\emptyset$, where$p_\emptyset$ is obtained as follows: The probability that a synapse has a specific neuron as presynaptic neuron is $1/N$, and hence, the probability that none of the NK/M process-local synapses has a particular neuron as presynaptic neuron is $p_\emptyset = (1 - 1/N)^{NK/M}$, where K is the number of incoming synapses per neuron and M is the total number of MPI processes.

1.2 Network and simulation parameters

Table 1, table 2 and table 3 summarize the network model and parameters.

**A: Model Summary**	
**Populations**	One
**Topology**	Ring topology
**Connectivity**	Nearest-neighbor (based on neuron GIDs) via gap junctions
**Neuron models**	Hodgkin-Huxley (HH)
**Channel models**	None
**Synaptic plasticity**	None
**External input**	Independent Gaussian white noise current to each neuron
**Measurements**	Total number of spikes generated per process
**Other**	None

**B: Populations**		
**Name**	**Elements**	**Size**
P	HH	N

**C: Connectivity**			
**Name**	**Source**	**Target**	**Pattern**
PP	P	P	Nearest-neighbor with K connections per neuron

Table 1: Tabular description of network model after Nordlie et al. (2009).

D: Neuron Model	
**Name**	HH
**Type**	Hodgkin-Huxley
**Membrane potential dynamics**	$$C_\mathrm{m}\dot{V}_i = -I_\mathrm{ionic}(V_i, m, h, n, p) + I_\mathrm{gap}$$ $$\begin{aligned} I_\mathrm{ionic} &= g_\mathrm{Na} m^3 h (V_i - V_\mathrm{Na}) \\ &+ (g_\mathrm{Kv3} p^2 + g_\mathrm{Kv1} n^4)(V_i - V_\mathrm{K}) \\ &+ g_\mathrm{leak}(V_i - V_\mathrm{leak}) \end{aligned}$$ $$\begin{aligned} \dot{m} &= \alpha_m(1-m) - m\beta_m \\ \dot{h} &= \alpha_h(1-h) - h\beta_h \\ \dot{n} &= \alpha_n(1-n) - n\beta_n \\ \dot{p} &= \alpha_p(1-p) - p\beta_p. \end{aligned}$$
**Synaptic current dynamics**	None

E: Synapse Model	
**Name**	Gap junction
**Type**	Birectional electrical coupling of membrane potentials
**Coupling**	Current into neuron j defined by: $I_{\mathrm{gap},ij}(t) = g_{ij}(V_i(t) - V_j(t))$

F: Input		
**Type**	**Target**	**Description**
**Gaussian noise**	P	Piecewise constant current with Gaussian distributed amplitude; amplitude sampled independently from $\mathcal{N}(\mu_\mathrm{noise}, \sigma_\mathrm{noise})$for each neuron in intervals of dt

Table 2: Tabular description of network model after Nordlie et al. (2009), continued.

	B: Populations	
**Name**	**Value**	**Description**
N	variable	Size of population P

	C: Connectivity	
**Name**	**Value**	**Description**
K	60	Number of gap junctions per neuron

	D: Neuron Model	
**Name**	**Value**	**Description**
–	–	see Mancilla et al. (2007)

	E: Synapse Model	
**Name**	**Value**	**Description**
g_{ij}	0.1 nS	Gap junction coupling strength

	F: Input	
**Name**	**Value**	**Description**
μ_{noise}	0 pA	Mean of noise current
σ_{noise}	250 pA	Standard deviation of noise current
dt	1.0 ms	Update interval of noise current

Table 3: Simulation parameters after Nordlie et al. (2009).

References

Mancilla, J. G., Lewis, T. J., Pinto, D. J., Rinzel, J., and Connors, B. W. (2007). Synchronization of Electrically Coupled Pairs of Inhibitory Interneurons in Neocortex. *J. Neurosci.* 27, 2058–2073

Nordlie, E., Gewaltig, M.-O., and Plesser, H. E. (2009). Towards reproducible descriptions of neuronal network models. *PLoS Comput. Biol.* 5, e1000456. doi:10.1371/journal.pcbi.1000456
